# A Novel Stress Echocardiography Pattern for Myocardial Bridge With Invasive Structural and Hemodynamic Correlation

**DOI:** 10.1161/JAHA.113.000097

**Published:** 2013-04-24

**Authors:** Shin Lin, Jennifer A. Tremmel, Ryotaro Yamada, Ian S. Rogers, Celina Mei Yong, Robert Turcott, Michael V. McConnell, Rajesh Dash, Ingela Schnittger

**Affiliations:** 1 Division of Cardiovascular Medicine, Stanford University School of Medicine, Stanford, CA (S.L., J.A.T., R.Y., I.S.R., C.M.Y., R.T., M.V.M.C., R.D., I.S.)

**Keywords:** angina, angiography, echocardiography, ischemia

## Abstract

**Background:**

Patients with a myocardial bridge (MB) and no significant obstructive coronary artery disease (CAD) may experience angina presumably from ischemia, but noninvasive assessment has been limited and the underlying mechanism poorly understood. This study seeks to correlate a novel exercise echocardiography (EE) finding for MBs with invasive structural and hemodynamic measurements.

**Methods and Results:**

Eighteen patients with angina and an EE pattern of focal end‐systolic to early‐diastolic buckling in the septum with apical sparing were prospectively enrolled for invasive assessment. This included coronary angiography, left anterior descending artery (LAD) intravascular ultrasound (IVUS), and intracoronary pressure and Doppler measurements at rest and during dobutamine stress. All patients were found to have an LAD MB on IVUS. The ratios of diastolic intracoronary pressure divided by aortic pressure at rest (Pd/Pa) and during dobutamine stress (diastolic fractional flow reserve [dFFR]) and peak Doppler flow velocity recordings at rest and with stress were successfully performed in 14 patients. All had abnormal dFFR (≤0.75) at stress within the bridge, distally or in both positions, and on average showed a more than doubling in peak Doppler flow velocity inside the MB at stress. Seventy‐five percent of patients had normalization of dFFR distal to the MB, with partial pressure recovery and a decrease in peak Doppler flow velocity.

**Conclusions:**

A distinctive septal wall motion abnormality with apical sparing on EE is associated with a documented MB by IVUS and a decreased dFFR. We posit that the septal wall motion abnormality on EE is due to dynamic ischemia local to the compressed segment of the LAD from the increase in velocity and decrease in perfusion pressure, consistent with the Venturi effect.

## Introduction

Myocardial bridges (MBs) are a common anatomic variant, predominantly involving the left anterior descending artery (LAD), with a prevalence of up to 85% on autopsy.^1^ Their anatomic identification is generally considered benign, although there is a small subset of patients with MBs who experience severe anginal symptoms and find relief with surgical unroofing.^2^

Identifying MBs among patients with angina is problematic. Historically, MBs have been found by coronary angiography (CA). However, the sensitivity of CA is very low (0.5% to 2.5%).^3–5^ Intravascular ultrasound (IVUS) can detect MBs with much greater sensitivity, both by systolic compression^6^ and a characteristic echo lucent “half‐moon” appearance^7^ in the tunneled vessel under the bridge. Tsujita et al^8^ found MBs in 23% of a group of 331 patients, all of whom underwent IVUS, as compared to 3% when using CA alone. Cardiac computed tomography (CT) is a promising noninvasive alternative with accuracy approaching that of IVUS.^9^ Nevertheless, a noninvasive functional test for clinically significant MBs would be valuable. Nuclear stress testing is not sensitive^10^ and the reported usefulness of exercise echocardiography (EE) in the detection of ischemia in MBs is near absent from the literature.

One challenge to functional testing is that the pathophysiology of how MBs can cause ischemia is poorly understood. There is the belief that MBs should not cause ischemia as they primarily affect only systolic and not diastolic flow. However, at high heart rates, diastole shortens and the systolic contribution to coronary blood flow increases. Also, the assumption has been that the compression of the MB during systole into diastole would have to limit blood flow distal to the MB during diastole to cause ischemia. Yet, in 12 symptomatic patients who were otherwise normal apart from harboring MBs, diastolic fractional flow reserve (dFFR) measured distal to the MB under dobutamine stress was <0.75 in only 5 patients.^11^

In this study, we describe a novel EE finding involving a focal abnormality in the end‐systolic to early‐diastolic septal wall motion with apical sparing. We initially had observed the mid‐septal buckling on exercise echocardiography in routine clinical care, with anecdotal cases of finding LAD MBs during follow‐up coronary angiography. We hypothesized that the focal EE finding represented ischemia caused by MBs under stress localized to the bridge.

To test our hypothesis we prospectively evaluated a cohort of patients with the EE finding using IVUS (as angiography alone is insensitive to detect MBs), and both intracoronary Doppler and pressure measurements during dobutamine stress, to quantify hemodynamic changes. The presence anatomically of an MB by IVUS and an abnormal dFFR within the MB would corroborate the echocardiographic finding and enhance our understanding of the potential role of MBs in patients with angina.

## Methods

### Study Population

All patients who underwent EE in the Stanford Hospital Echocardiography Laboratory between April 2011 and March 2012 were considered for this study. Patients with late‐systolic/early‐diastolic buckling of the septum with apical sparing on EE (details given below) and symptoms of moderate‐to‐severe angina—either typical or atypical—were prospectively enrolled in the invasive study. Patients with acute coronary syndrome or previously known MBs were excluded because our aim was to conduct a prospective evaluation of the EE finding. All patients gave informed consent. The study was approved by Stanford University Institutional Review Board.

### Echocardiogram

**Figure 1.**
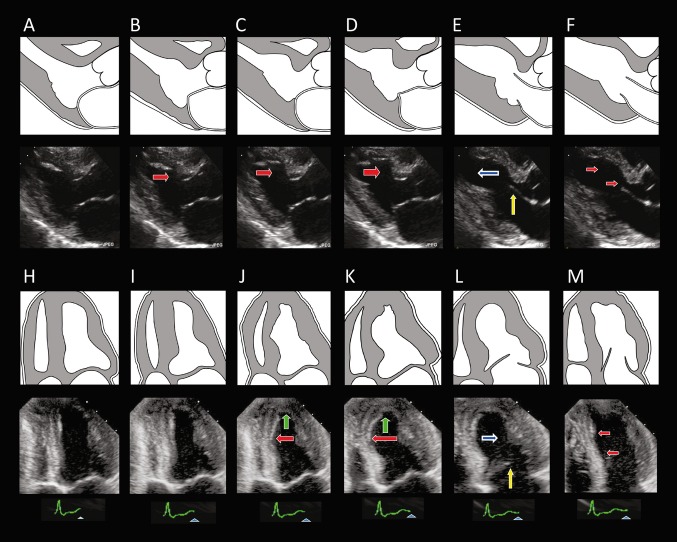
Myocardial bridge (MB) stress echocardiographic findings with schematic graphics. Six consecutive frames demonstrate the increasing to decreasing degree of buckling in the middle segment of the septum during end‐systole to early‐diastole in the parasternal long‐axis (A through F) and corresponding apical 4‐chamber (H through M) views. A, Normal antero‐septal motion in mid‐systole. B, Mild antero‐septal buckling (red arrow) at the peak of the T‐wave in the ECG. C, Further progression of the mid antero‐septal buckling (larger red arrow). D, Peak antero‐septal buckling (largest red arrow) occurs with the mitral valve still closed. E, The mid antero‐septal segment starts to relax, moving toward the center of the cavity (blue arrow) and the mitral valve opens (yellow arrow). F, Further movement of the mid antero‐septal segment toward the center of the ventricle along with a global expansion of the cavity (dual red arrows). H and I, Normal septal motion in mid to late systole. J, Mild septal buckling (red arrow) at the early down‐slope of the T‐wave in the ECG. Note that the apical region does not dilate, on the contrary it gets smaller (green arrow), consistent with normal contraction, while the septum buckles. K, Peak septal buckling (larger red arrow) occurs with the mitral valve still closed. Again, note that the apical cavity continues to get smaller (green arrow) while the septum buckles. L, The mid septal segment starts to relax (blue arrow) and mitral valve opens (yellow arrow). M, Further relaxation of the mid septal segment along with a global expansion of the cavity (dual red arrows).

In routine clinical care, we had previously noticed a characteristic EE wall motion abnormality associated with MBs of the LAD, as detected by CA. During the end‐systolic to early‐diastolic phase, we identified a focal buckling of the septum with apical sparing (Figure 1). These findings can be visualized most prominently upon analysis of image loops frame‐by‐frame. The focal buckling is most apparent in the mid‐antero–septal wall in the parasternal long‐axis and apical 3‐chamber views. It can also be seen in the mid‐septum in the 4‐chamber view. The apical sparing is best seen in the latter. A representative echo loop of the parasternal long view may be accessed in the accompanying video. None of the patients had “typical” wall motion abnormalities extending to the apex or anterior wall to suggest a fixed LAD coronary artery stenosis.

EE was performed according to laboratory standards, using an iE33 Ultrasound system (Philips Healthcare). An average of 5 to 10 beats per loop was recorded at rest and post stress. Images were obtained in the parasternal long and short axis, apical 2‐, 4‐, and 3‐chamber views and analyzed on an offline Xcelera work station at normal speed, in slow motion and frame‐by‐frame by a senior echocardiographer (I.S.). All patients performed the Bruce treadmill protocol^12^ test with a goal of reaching a target heart rate of 85% of maximal predicted for age. A 12‐lead ECG was obtained at baseline and during the test.

### Catheterization Procedure

Oral negative chronotropic agents were discontinued 2 days prior to the procedure. Baseline CA was obtained before and after 200 μg of intracoronary (IC) nitroglycerine (NTG), in multiple standard views, using the Advantx fluoroscopy system (General Electric Company) with acquisitions made at 15 frames per second. IC NTG was administrated to make the bridge more apparent angiographically.^13^

IVUS was performed with a 40‐MHz mechanical transducer ultrasound catheter (Atlantis SR Pro2, Boston Scientific Corp) in the LAD with placement of the IVUS sensor as far distally as possible in the vessel. Both automatic and manual pullbacks were performed.

The presence of an MB was defined by IVUS, either by the identification of an echo lucent half‐moon sign or by systolic compression (≥10% systolic compression during the cardiac cycle).^8^ The echo lucent half‐moon sign was identified during live recording, whereas percent systolic compression was done offline. Only those patients with an echo lucent half‐moon sign underwent the dobutamine stress protocol. Patients without the IVUS echo‐lucent half‐moon sign were not studied with dobutamine as the procedural delay to allow offline analysis for MB confirmation could not be justified ethically for this study.

Hemodynamic measurements were made with the ComboWire XT Pressure and Flow Wire (Volcano). Pressure and flow velocity waveforms were taken before and at peak dobutamine stress. Pressure and flow velocities were measured at least 1 cm proximal to the MB, within the MB, and at least 1 cm distal to the MB. Dobutamine was given intravenously in increments of 10 μg kg^−1^ min^−1^ every 3 minutes until the maximal heart rate on previous EE was achieved, a maximal dose of 50 μg kg^−1^ min^−1^ with up to 1.0 mg atropine was administered, or the patient developed ischemic symptoms.

### Data Analysis

Quantitative coronary angiography (QCA) was performed using the computer‐assisted method QAngio XA7.3 (Medis). The measurements of interest include the diameters of the reference and tunneled vessels during diastole and systole after administration of IC NTG in left anterior oblique (LAO) and right anterior oblique (RAO) cranial views. The boundaries of the tunneled vessel by QCA were confirmed by comparisons with the IVUS data.

IVUS images were stored onto DVD for offline analysis. Maximum percent systolic compression was calculated by echoPlaque software (Indec Systems, Inc) and was defined as the change in vessel area during the cardiac cycle divided by vessel area during diastole, using a single IVUS frame during manual pullback. An ECG was recorded simultaneously. The maximal plaque burden (MPB) was measured as the difference between vessel and lumen areas divided by vessel area with the largest plaque burden. IVUS length was measured from the first proximal appearance of the half‐moon sign to its distal end, or for those without the sign, the length of the compressed region.

Combowire recordings were stored on the ComboMap console for offline analysis. Using a digital electronic caliper, instantaneous Pd/Pa (the fraction of diastolic coronary artery pressure divided by diastolic aortic pressure) at rest and with stress were measured throughout diastole 3 to 5 times per beat and averaged using 2 to 3 beats, to arrive at an average Pd/Pa (at rest) and average dFFR (with stress). The peak Doppler velocity was recorded for each site at rest and with stress by averaging the peak velocities over 3 beats.

### Statistical Analysis

For all continuous variables, mean and standard deviations were calculated. Hemodynamic measurements within the MB were compared to proximal and distal measurements, at rest and with stress using the paired Student *t*‐test. Confidence intervals (CIs) were derived from the *t*‐test analysis. As normality of the data is difficult to ensure with small sample sizes, a nonparametric analysis was also performed with the Wilcoxon signed‐rank test. A *P*‐value of <0.05 was considered significant.

## Results

### Patient Characteristics

Eighteen patients (age 16 to 62 years, median 43 years) with angina and EE evidence of septal wall motion abnormality at end‐systole to early‐diastole with apical sparing (Figure 1) were consecutively enrolled in the invasive procedure. Our cohort had few coronary artery disease (CAD) risk factors (Table 1). Twelve of 18 patients had reproducible symptomatology during EE. The median work load reached was 12 metabolic equivalents (METS) with median stress time of 10 minutes. Four individuals had 1 mm or more ST segment depression at peak stress.

**Table 1.** Characteristics of the Study Group

Subject Number	Age, y/Sex	Risk Factors for CAD*	Angiographic MB Without NTG/With NTG	Angiographic Compression Diastole/Systole (%)	IVUS Compression (%)	IVUS Half‐Moon Sign	IVUS MB Length (mm)	IVUS Proximal LAD Maximum Plaque Burden (%)	Number of Intrabridge Septals on IVUS	Peak HR During Stress	Peak Dobutamine (μg kg^−1^ min^−1^)/Atropine Dose (mg)
1	27F	2 Pack years	−/−	31.2/54.0	29	+	14.4	39.1	1	163	40/0.8
2	47M	HL, 2 pack years, age	−/−	34.7/52.7	35	+	25.8	35.5	1	129	40/0.4
3	46F	4 Pack years	−/+	35.8/28.0	18	+	5.4	43.2	1	150	20/−
4	40M	HL	+/+	14.8/71.7	39	+	34.7	44.8	4	156	50/0.2
5	45F	None	−/−	23.4/34.6	21/17*	+	18.7/8.3*	26.8	2	164	50/0.4
6	62F	HL, 25 pack years, age	−/−	6.5/43.7	52	+	29.7	59.7	2	126	50/0.8
7	16F	None	−/−	21.7/22.7	20	−	N/A	22.7	N/A	N/A	−
8	26M	None	−/+	9.8/51.8	31	+	46.2	15.2	2	163	50/0.4
9	41M	None	−/+	18.5/36.9	42	+	36.0	52.6	2	150	50/0.4
10	38F	None	−/+	17.7/37.1	13/18*	+	14.9/9.9*	25.7	3	169	50/0.2
11	20F	HTN	+/+	17.8/47.1	39	+	25.0	20.1	3	168	50/0.4
12	19F	None	−/−	31.0/20.3	25	−	N/A	15.7	N/A	N/A	−
13	55F	HTN, 5 pack years, age	−/−	7.3/34.1	30.5	+	>24.5	20.0	2	141	50/1.0
14	53F	HL	+/+	22.8/45.2	24/19*	+	16.5/9.5*	39.0	3	154	50/0.4
15	58M	Age	−/−	36.5/58.5	39	+	15.5	47.8	2	154	40/0.6
16	46F	None	−/−	1.0/19.3	23	−	N/A	33.7	N/A	N/A	−
17	45F	None	+/+	18.8/5.4	16	+	>17	16.0	1	N/A*	−
18	33F	None	+/+	34.2/64.3	12	+	8.0	16.0	2	171	50/0.4

CAD indicates coronary artery disease; MB, myocardial bridge; NTG, nitroglycerine; IVUS, intravascular ultrasound; LAD, left anterior descending artery; HR, heart rate.

* Risk factors include hyperlipidemia (HL), tobacco use (pack years), diabetes, hypertension (HTN), age, and family history.

* Multiple bridges detected.

* Equipment failure.

### Coronary Angiogram and QCA Findings

No patient had visually significant CAD on CA. An MB in the LAD could be seen at rest in only 5 individuals with an additional 4 apparent after administration of IC NTG. By QCA, the mean percent diameter stenosis was 21.3±2.6% during diastole and 40.4±4.1% during systole (Table 1).

### IVUS Findings

**Figure 2.**
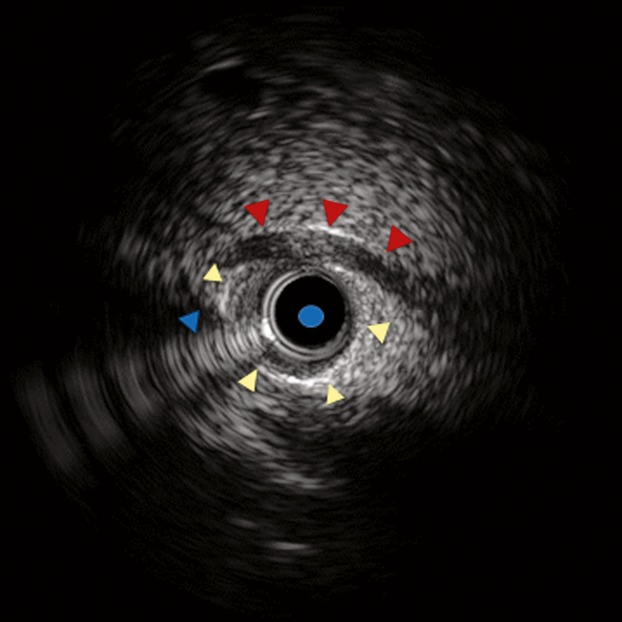
Intravascular ultrasound (IVUS) picture of an echo‐lucent half‐moon sign. The red arrows outline the echo‐lucent half‐moon sign. The yellow arrows outline the vessel wall. The blue round circle is positioned in the center of the IVUS catheter and the blue arrow points to an artifact from the catheter.

IVUS revealed the pathognomonic half‐moon sign for MBs (Figure 2) in 15 of 18 patients. Though specific, this feature is not 100% sensitive, as the compression of MBs has been detected in the absence of the half‐moon finding.^13^ In the 3 patients without the half‐moon sign on IVUS, each had a systolic compression of the LAD at rest of 20%, 23%, and 25% by IVUS. Maximal compression occurred in end systole, with delayed opening in early‐diastole, making the finding unlikely to be an artifact in vessel lumen area related to the IVUS catheter pull back. For all 18 patients, mean maximum arterial compression was 28.2±2.5%, and mean MB length was 23.7±2.8 mm. Three patients had 2 MBs. Notably, all patients had at least one visible septal branch within the region of the MB. Two patients had MBs that extended beyond the distal extent to which the IVUS could be safely advanced.

All 18 patients had coronary plaque (mean MPB, 33.8±3.2%) seen on IVUS in the proximal LAD, preceding the entrance to the tunneled vessel. The MPB was found at a mean distance of 29.8±3.2 mm proximal to the entrance of the tunnel, with 13 of 15 patients also having plaques of at least 15% within 20 mm preceding the tunnel. The lesions were not visualized on CA. The 3 patients without the half‐moon sign had a MPB of 16% to 34% proximal to the area of maximal compression of the LAD, including the 2 youngest patients (Table 1).

### Hemodynamics

Of 15 patients with the half‐moon sign on IVUS, one was excluded because of equipment malfunction. For 2 patients, only proximal and intrabridge measurements could be obtained due to the LAD anatomy—distal measurements could not be made in one of the patients because the LAD tapered to a small‐caliber vessel preventing accurate readings, and in the other the MB extended to the apex (Table 1). Thirteen of 14 patients studied with dobutamine stress reported symptoms of angina at peak dobutamine infusion, reproducing their clinical symptoms.

**Figure 3.**
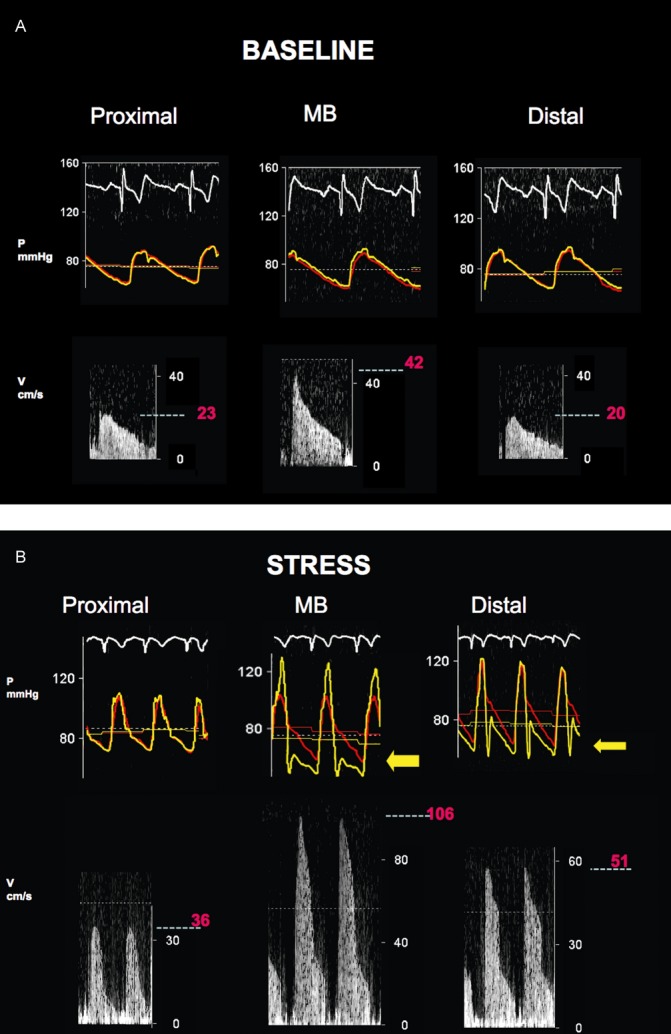
Representative readings of flow velocity and pressure. At baseline (A) Doppler flow velocity and pressure data are shown for one representative patient proximal, within, and distal to the myocardial bridge (MB). Pressure in the aorta (red pressure line) vs the coronary artery (yellow pressure line). Doppler velocity profiles show maximum peak velocity readings at rest of 23, 42 (with prominent “fingertip”), and 20 cm/s, respectively. With peak stress (B), there is no significant change in the 2 pressure lines proximally. Intrabridge (MB) there is systolic overshoot (yellow line) and diastolic decline in pressure, resulting in a diastolic fractional flow reserve (dFFR) of 0.74 (large yellow arrow). Distal to the bridge, there is diastolic pressure recovery with a dFFR of 0.88 (small yellow arrow). Stress Doppler peak velocities are 36, 106, and 51 cm/s, respectively.

Representative pressure and Doppler flow velocity curves (Figure 3) show, at rest, the characteristic MB “fingertip” Doppler pattern, but no difference in aortic and coronary pressure, while with peak dobutamine they show a marked rise in velocity and drop in intracoronary pressure within the MB, with partial recovery distal to the MB.

**Figure 4.**
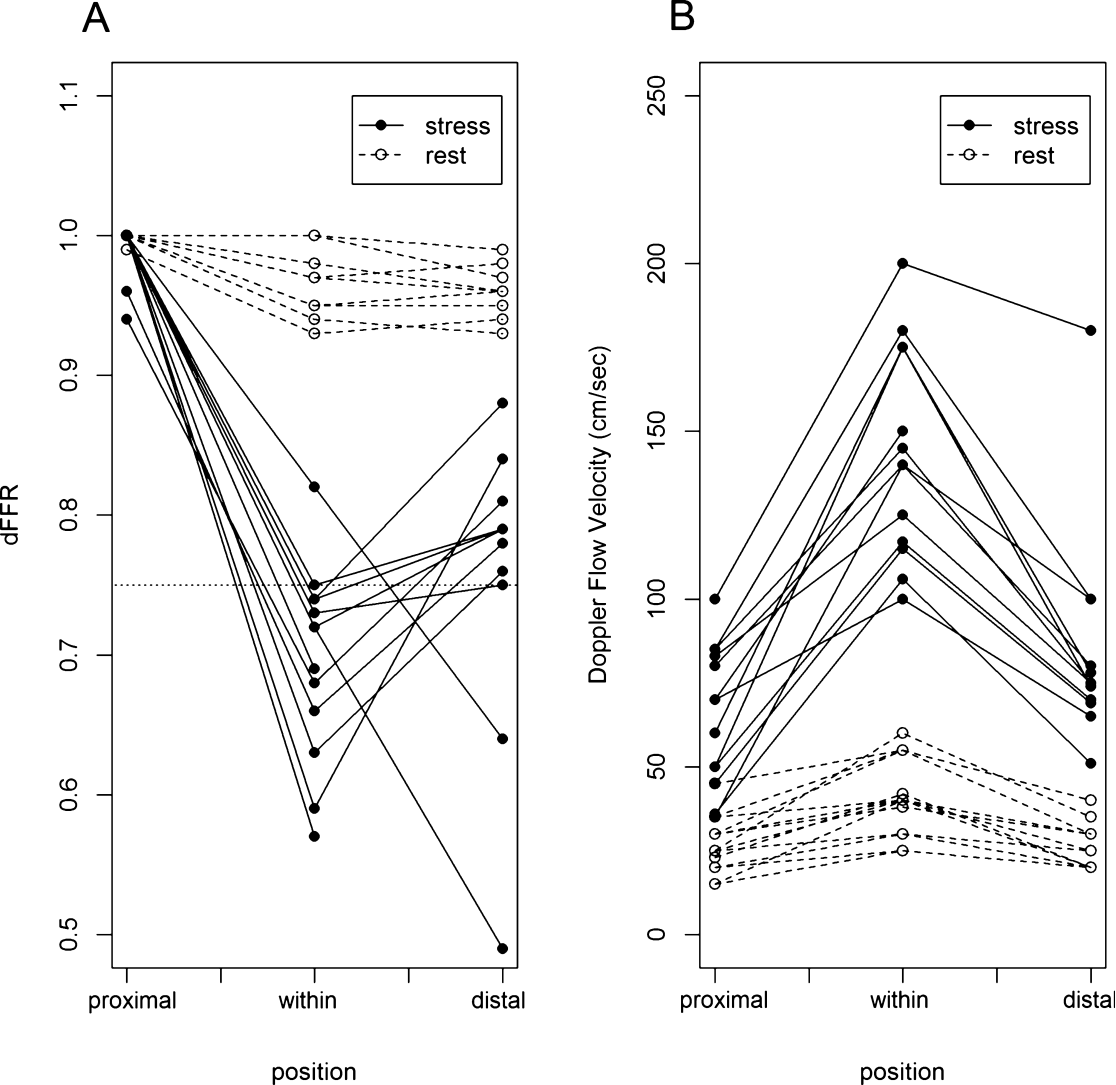
Hemodynamics of MB patients. Measured Pd/Pa at rest and dFFR with stress proximal, within, and distal to the bridge are plotted in (A). The threshold of 0.75 is shown as a dashed line. Measured peak velocity at rest and with stress proximal, within, and distal to the bridge are plotted in (B). Values corresponding to rest and stress are demarcated differently. MB indicates myocardial bridge; Pd/Pa, diastolic intracoronary pressure divided by aortic pressure at rest; dFFR, diastolic fractional flow reserve.

Quantitative analysis showed that the baseline Pd/Pa ratio proximal to, within, and distal to the MB were near 1.0 (Figure 4A, Table 2). With peak stress, however, all 14 patients with MBs studied hemodynamically had significantly abnormal dFFRs (≤0.75) within the MB, distal to it, or both. Of these, 13 of 14 had low dFFRs within the MB and out of 12 patients with obtainable distal measurements, 3 had a low distal dFFR (Figure 4A, Table 2). One of these 3 patients had only a distal dFFR below threshold; the other 2 had low dFFRs both within the MB and distal to it. It is noteworthy that these 3 patients with an abnormal distal dFFR had the longest MBs in our cohort, measuring 35 mm and 46 mm, with the third patient having 2 serial MBs. It is also important to note that with stress, dFFRs remained near 1.0 in the normal coronary segment proximal to the MB.

**Table 2.** Hemodynamics

Subject Number	Pd/Pa and dFFR									Velocity								
	Rest			Stress			% Change From Rest to Stress			Rest (cm/s)			Stress (cm/s)			% Change From Rest to Stress		
	Prox	Within	Distal	Prox	Within	Distal	Prox	Within	Distal	Prox	Within	Distal	Prox	Within	Distal	Prox	Within	Distal
1	1.00	0.95	0.95	1.00	0.59	0.84	0	−38	−12	35	40	30	60	175	74	71	3.4e2	1.5e2
2	1.00	0.97	0.98	1.00	0.74	0.88	0	−24	−10	23	42	20	36	106	51	57	1.5e2	1.6e2
3	1.00	1.00	0.99	1.00	0.74	0.88	0	−26	−11	30	38	30	50	117	70	67	2.1e2	1.3e2
4	0.99	0.93	0.94	1.00	0.73	0.75	1	−22	−20	30	55	30	83	125	75	1.8e2	1.3e2	1.5e2
5	1.00	0.98	0.96	1.00	0.63	0.76	0	−36	−21	25	30	25	85	145	75	2.4e2	3.8e2	2.0e2
6	1.00	0.95	0.96	1.00	0.72	0.79	0	−24	−18	45	55	40	60	175	78	33	2.2e2	95
8	1.00	0.97	0.96	1.00	0.82	0.64	0	−15	−33	30	40	25	100	200	180	2.3e2	4.0e2	6.2e2
9	1.00	1.00	0.97	1.00	0.69	NA	0	−31	NA	20	25	20	70	150	NA	2.5e2	5.0e2	NA
10	1.00	0.94	0.93	1.00	0.75	0.79	0	−20	−15	20	30	20	70	100	65	2.5e2	2.3e2	2.3e2
11	1.00	0.95	0.96	1.00	0.74	0.79	0	−22	−18	15	40	20	85	180	100	4.7e2	3.5e2	4.0e2
13	1.00	0.97	0.96	1.00	0.57	NA	0	−41	NA	35	55	NA	50	175	NA	43	2.2e2	NA
14	1.00	1.00	0.97	1.00	0.72	0.49	0	−28	−49	25	60	35	35	140	100	40	1.3e2	1.9e2
15	1.00	0.94	0.93	0.94	0.68	0.81	−6	−28	−13	15	25	20	45	115	69	2.0e2	3.6e2	2.5e2
18	1.00	0.95	0.96	0.96	0.66	0.78	−4	−31	−19	25	40	30	80	140	80	2.2e2	2.5e2	1.7e2

Pd/Pa indicates diastolic intracoronary pressure divided by aortic pressure at rest; dFFR, diastolic fractional flow reserve; Prox, proximal.

At rest, the average peak velocity measurements proximal to, within, and distal to the MB were 26.8±2.2, 41.1±3.1, and 26.5±1.8 cm/s, respectively (Figure 4B, Table 2). The peak velocity within the MB was significantly increased compared to readings made proximally (mean difference 14.4 cm/s, 95% CI: 9.2 to 19.7, *P*=5 × 10^−5^) and distally (mean difference 13.5 cm/s, 95% CI: 9.0 to 18.0, *P*=3 × 10^−5^). The velocity contour within the MB had a “fingertip” shape in all patients, characteristic for MBs.^7^ With stress, there was an increase in Doppler flow velocity at all positions. Average peak diastolic flow velocities proximal to, within, and distal to the MB were 64.9±5.4, 145.9±8.3, and 84.8±9.5 cm/s, respectively. Again, with stress, the peak velocity within the MB was significantly increased compared to readings made proximally (mean difference 81.0 cm/s, 95% CI: 64.3 to 97.7, *P*=1 × 10^−7^) and distally (mean difference 58.4 cm/s, 95% CI: 42.8 to 74.0, *P*=5 × 10^−6^). More importantly, the degree of the increase of peak velocity in the MB compared to the average of proximal and distal values was greater at stress than at rest (mean difference 53.5 cm/s, 95% CI: 38.2 to 68.7, *P*=9 × 10^−6^). All these comparisons remained significant by nonparametric analysis (all *P*<0.003). In viewing the dFFRs and peak velocities of all 14 subjects upon stress, an inverse relationship between the 2 parameters can be seen at each of the 3 positions sampled. Consistent with this finding, the 3 lowest distal dFFRs corresponded to the 3 highest distal peak velocities (Figure 4B).

## Discussion

In our study of symptomatic patients with a distinctive focal wall motion abnormality upon EE, we invasively confirmed the presence of an MB by IVUS, by identification of an echo lucent half‐moon sign and/or systolic compression, and also demonstrated significant hemodynamic disturbances associated with the MB, including a marked increase in flow velocity and an abnormal dFFR during dobutamine stress. Also, all patients had at least one septal branch within the MB segment in concordance with a focal abnormality seen on EE. This is the first time, to our knowledge, that a noninvasive functional abnormality for the identification of an MB has been identified and undergone extensive invasive correlation in patients with anginal symptoms.

In previous work, Escaned et al^11^ found that dFFR under dobutamine stress measured distal to the bridge was abnormal only in a minority of patients with angiographically confirmed MBs, leaving the question of why an abnormal value of this hemodynamic measurement of ischemia was not found more consistently in this cohort of symptomatic patients. Our observation of a focal, end‐systolic/early‐diastolic buckling of the septum with apical sparing on EE, in a similar patient population, led to the hypothesis that the hemodynamic disturbance and, thereby, ischemia was local to the MB rather than distal to it.

There is literature supportive of this concept. Klues et al^14^ measured diastolic blood pressure proximal to, within, and distal to the MB in 12 patients in the resting state and found a trend toward lower diastolic pressure intrabridge with pressure recovery distally. This hemodynamic change in the Pd/Pa ratio should be exacerbated under dobutamine stress within the bridge.

**Figure 5.**
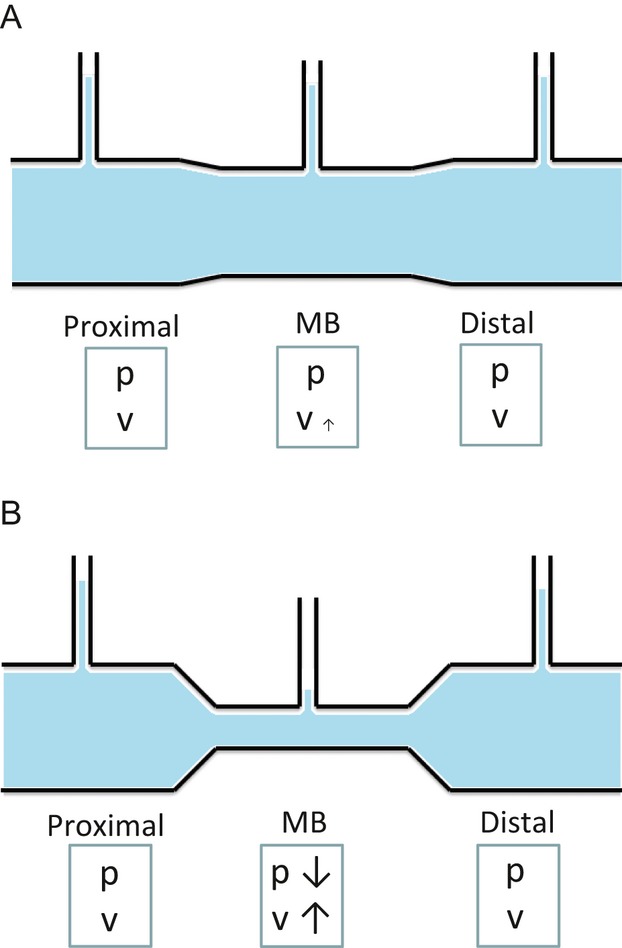
Venturi effect. With mild constriction (A), there is little change in the pressure and velocity throughout the pipe, with only a small increase in velocity and drop in pressure within the myocardial bridge (MB) segment. With marked constriction (B), velocity increases and the pressure decreases within the narrowed MB region. Note the water levels of the vertical tubes depicting the pressure drop and recovery. We posit that during end‐systole and early‐diastole upon stress, the MB causes the left anterior descending artery (LAD) to take on the shape of the depicted pipe and thereby the corresponding intrabridge pressure drop as well.

The most common finding in the patients in our cohort was an abnormal dFFR that was present only within the bridge. We posit a model of ischemia involving the Venturi effect (Figure 5). During end‐systole to early‐diastole, the tunneled portion of a vessel resembles the constricted section of a pipe. The laws governing fluid dynamics dictate that the fluid velocity must increase when it passes through a narrowed area to satisfy the principle of continuity, while pressure must decrease to satisfy the principle of conservation of energy by Bernoulli's equation. The Venturi effect may be derived from a combination of these 2 concepts. Thus, we infer that the decrease in the pressure in the tunneled section, and thereby the perfusion pressure to the corresponding septal branch vessels, leads to focal ischemia. Conversely, distal to the bridge, the vessel area increases, resulting in a decrease in velocity and pressure recovery, and hence, resulting in a nonischemic distal dFFR. This Venturi effect serves as a model to explain the EE finding of a focal septal wall motion abnormality with apical sparing supported by our hemodynamic findings of a pressure drop in the MB, during dobutamine stress.

For the 3 subjects in our study who had an abnormal distal dFFR, 2 had the longest and second longest MBs, and the other harbored 2 serial MBs. It is possible that the length of arterial compression increased with dobutamine stress, a phenomenon reported previously by Escaned et al,^11^ thereby possibly obscuring the true velocity and pressure measurements distal to the long and serial bridges. We hypothesize that we were simply not able to extend the distal pressure measurement far enough to be truly outside of the MB during stress in these 3 cases.

Another corroborative observation of the timing of the end‐systolic to early‐diastolic wall motion abnormality is found in the cross sectional area tracings at the site of compression by IVUS, noted in this study as well as in another report.^6^ The smallest luminal area of the MB is in late‐systole and very early‐diastole, hence this is when flow is most restricted. The timing of this area change differs from a fixed stenosis, in which the area does not change between systole and diastole. Also, in normal coronary physiology, the proportion of coronary flow during systole increases with an elevated heart rate, to compensate for the decrease in diastolic filling time.^15^ With a lumen area reduction at end‐systole and early‐diastole in MBs, this adaptive mechanism for maintenance of adequate blood flow with exercise may be compromised.

Jhi et al recently reported a finding on speckle‐tracking strain echocardiography of a “bi‐phasic” or “double‐peak” radial strain with dobutamine stress associated with MBs.^16^ We believe that the septal buckling we have described on EE corresponds temporally to the late systolic dip in the radial strain described by Jhi et al although their invasive confirmation of MB was by CA alone. The mechanism behind this biphasic wall motion and radial strain are not known and will require further investigation. We speculate that maximum systolic compression of the MB in late systole, followed by relaxation in diastole, is related to these phenomena.

### Limitations

The major limitation was the lack of a control group in this proof‐of‐concept study. We could not justify these extensive invasive measurements in patients with normal EE. Thus, sensitivity, specificity, and other standard measures for validating a putative test were not estimated. These parameters will need to be established before our echocardiographic findings can be widely adopted for routine clinical care. Additionally, the sample size of this cohort was relatively small and 3 patients without the IVUS half‐moon echo‐lucent sign were not studied with dobutamine as the procedural delay to allow offline analysis for MB confirmation could not be justified for this study. Furthermore, it may not be possible to reach the distal LAD in long MBs with small distal segments. Also, with stress the optimal definition of the Doppler signal can be challenging to record when the patient is in pain and is tachycardic. Finally, we do recognize that because the constriction may not be uniform within all bridges and can change with time, there could be dynamic changes in velocity and pressure, requiring more complex modeling for further mechanistic validation.

## Conclusion

We have identified a novel EE finding, consisting of a transient focal buckling in the end‐systolic to early‐diastolic motion of the septum with apical sparing which correlates prospectively with the presence of LAD MB by IVUS, in a cohort of symptomatic patients. All patients with invasive hemodynamic measurements under dobutamine stress demonstrated an abnormal dFFR within and/or distal to the MB, consistent with MB‐related ischemia. Finally, we found that our pressure and Doppler flow results support the concept that the Venturi effect may underlie the focal abnormalities seen during noninvasive and invasive functional testing.

The potential to detect physiologically important myocardial bridging noninvasively using exercise echocardiography is promising, and we hope this report will encourage other physicians and investigators to consider further evaluation of patients with angina and suspected MBs.
